# Aircraft Sensor Fault Diagnosis Based on GraphSage and Attention Mechanism

**DOI:** 10.3390/s25030809

**Published:** 2025-01-29

**Authors:** Zhongzhi Li, Jinyi Ma, Rong Fan, Yunmei Zhao, Jianliang Ai, Yiqun Dong

**Affiliations:** 1 Department of Aeronautics and Astronautics, Fudan University, Shanghai 200433, China; zzli22@m.fudan.edu.cn (Z.L.); jyma21@m.fudan.edu.cn (J.M.); rfan24@m.fudan.edu.cn (R.F.); aijl@fudan.edu.cn (J.A.); 2 School of Aerospace Engineering and Applied Mechanics, Tongji University, Shanghai 200092, China; yunmeizhao@tongji.edu.cn

**Keywords:** aircraft sensors, fault diagnosis, graph neural network, attention mechanism

## Abstract

Aircraft sensors are crucial for ensuring the safe and efficient operation of aircraft. However, these sensors are vulnerable to external factors that can lead to malfunctions, making fault diagnosis essential. Traditional deep learning-based fault diagnosis methods often face challenges, such as limited data representation and insufficient feature extraction. To address these problems, this paper proposes an enhanced GraphSage-based fault diagnosis method that incorporates attention mechanisms. First, signal data representing the coupling characteristics of various sensors are constructed through data stacking. These signals are then transformed into graph data with a specific topology reflecting the overall sensor status of the aircraft using K-nearest neighbor and Radius classification algorithms. This approach helps fully leverage the correlations between data points. Next, node and neighbor information is aggregated through graph sampling and attention-based aggregation methods, strengthening the extraction of fault features. Finally, fault diagnosis is performed using multi-layer aggregation and transformation within fully connected layers. Experiments demonstrate that the proposed method outperforms baseline approaches, achieving better detection performance and faster computational speed. The method has been validated on both simulated and real-flight data.

## 1. Introduction

Aerospace sensors are used to measure flight parameters such as airspeed and angles, playing a crucial role in ensuring the normal and safe operation of aircraft [1,2]. However, sensors measuring parameters like airspeed and angle of attack (AOA) are installed on the external surface of the aircraft, where they are directly exposed to the atmospheric environment, making them susceptible to influences such as rain, frost, and icing. While sensors measuring attitude angles are installed inside the aircraft fuselage, they remain sensitive to environmental factors such as temperature and humidity. These sensors are prone to malfunctions, which can affect the aircraft’s performance. Therefore, the development of fault diagnosis technology for aerospace sensors is essential to ensure safe aircraft operations [3,4].

Traditional fault diagnostic methods mainly involve two steps: feature extraction using signal processing methods and fault classification or regression using machine learning techniques [5,6]. Wang et al. [7] proposed an actuator fault diagnosis scheme for flight control systems based on model identification techniques. The approach combined system identification through a linear model, employing a closed-loop subspace model identification algorithm, demonstrating higher fault diagnosis accuracy. He et al. [8] proposed a nonlinear disturbance observer-based approach for aircraft fault diagnosis by making use of dynamic and kinematic relations of the aircraft. Dewallef et al. [9] proposed a diagnostic method for aircraft engines that integrates a soft-constrained Kalman filter, which enhances the estimation of unknown health parameters. Marcos et al. [10] presented a H∞-based fault diagnostic methods for the longitudinal motion of the Boeing 747-100/200 aircraft. Closed-loop simulations with a high-fidelity nonlinear model in the presence of gust and noise were performed to validate the performance of the proposed scheme. Cartocci et al. [11] presented a data-driven fault diagnosis scheme for aircraft sensors using PCA and D-PCA techniques. The method integrated evidence-based filtering to enhance fault isolation, demonstrating effectiveness in reducing false alarms. Heredia et al. [12] presented a sensor fault detection and diagnosis system for small autonomous helicopters based on analytical redundancy. The system has been tested with real helicopter flight data, yielding promising performance. However, these traditional fault diagnosis methods rely on models of aircraft dynamics and sensor characteristics such as delay, which are challenging to accurately identify. Additionally, the need for extensive parameter tuning due to external disturbances has limited the further application of traditional fault diagnosis algorithms.

With the rise of deep learning (DL) theories and computational resources, intelligent technologies have made significant progress in feature extraction [13]. Many powerful deep neural networks (DNNs) have been developed, such as convolutional neural networks (CNNs) [14,15], autoencoders (AEs) [16,17], and recurrent neural networks (RNNs) [18,19]. These models have also been successfully applied in fault diagnosis. Wei et al. [20] proposed an offline diagnosis method of CNN with novel topology. Simulation results show that the proposed method can accurately diagnose the actuator fault and its position sensor. Toma et al. [21] introduced a framework combining deep autoencoders and convolutional neural networks for bearing fault classification in induction motors. The proposed approach effectively identifies faults by automatically extracting and classifying signal features. Yang et al. [22] developed a multi-head deep neural network based on sparse autoencoders for both diagnostics and the detection of unknown defects, enhancing the flexibility and accuracy of diagnostics. Ma and Mersha [23] explored a data-driven approach using recurrent neural networks (RNNs) for fault detection in AOA sensors in aircraft, providing a robust framework for handling aerospace sensor anomalies. Although these DNN-based methods effectively capture hidden features in conventional data (e.g., time series), they face inherent limitations in processing multi-sensor aircraft data. Most methods overlook two critical types of interdependencies: the coupling relationships between different sensors, and the correlations between different aircraft states characterized by combinations of sensor measurements. When standard convolution operations are performed on multi-sensor measurements, they simply take a weighted sum of the sensor readings with corresponding convolution kernels, without considering these complex interdependencies. To address this issue, an increasing number of applications now represent data as irregular graphs, where relationships between different states can be naturally modeled through edges and their weights. However, the complexity of such graph data poses critical challenges for standard DNN-based methods, making some essential operations (e.g., convolution) easy to apply in Euclidean domains but difficult to model in non-Euclidean spaces.

In recent years, graph neural networks (GNNs) have emerged as a novel type of neural network designed for modeling graph data [24,25]. Inspired by concepts from DL, such as CNNs, RNNs, and AEs, new definitions have been extended to complex graph data, resulting in corresponding graph convolutional networks (GCNs) [26], graph recurrent neural networks (GRNNs) [27], and graph autoencoders (GAEs) [28]. These neural networks have been successfully implemented across various domains, including chemistry, commonsense reasoning, natural language processing, social networks, and traffic flow prediction [29]. Recently, researchers have increasingly applied GNNs to fault diagnosis due to their ability to model interdependencies between data and embed these into extracted features. For example, Shi et al. [30] proposed a novel unsupervised multivariate time series anomaly detection framework based on GCNs, which simultaneously models the correlations between variables and the importance of variables at each time period. Xie et al. [31] proposed an anomaly detection method for aerospace data based on graph neural networks. The proposed method was applied to convert linear structure data into graph data, showing good effectiveness and robustness. Xiao et al. [32] presented a control area network graph attention networks (CAN-GAT) model to implement the anomaly detection of in-vehicle networks. The CAN-GAT model claimed improved accuracy among the compared baseline methods, and has good detection speed performance. Qiu et al. [33] proposed a reinforced graph regularization fault diagnosis network to address the difficulty in fusing multiple data sources and the insufficient consideration of sample correlations. The proposed approach was validated using a high-speed aviation-bearing dataset, showing promising performance. However, existing GNN-based methods have not fully explored the potential of combining different graph construction strategies and attention mechanisms for fault feature extraction. Moreover, their applications in fixed-wing aircraft sensor fault diagnosis remain limited, where the challenge lies in processing data from multiple heterogeneous sensors operating under complex flight conditions.

To address the above issues, this paper proposes a multi-sensor graph convolutional fault diagnosis method based on the combination of attention mechanisms and the GraphSage network. The highlights of this paper are summarized as follows:

1. Multi-Sensor Data Stacking and Graph Construction: This approach effectively integrates data from multiple sensors using data stacking techniques and leverages KNN and Radius algorithms to generate graph structures. The resulting graph captures diverse and comprehensive fault information from various sensors, enhancing the accuracy and robustness of fault diagnosis classification.

2. Enhanced Fault Feature Extraction via Attention Mechanisms: The model incorporates attention mechanisms to transform both node attributes and sampled neighbor node features, significantly improving the model’s ability to identify and learn relevant fault features.

3. Multi-Layer Aggregation and Validation on Diverse Datasets: The fault diagnosis is achieved through multi-layer information aggregation and feature transformation. The proposed method is rigorously validated on both simulated and real-world data, demonstrating its superior performance and high fault diagnosis detection rate compared to other advanced methods across diverse datasets.

The remainder of this paper is organized as follows: Section 2 formally defines the fault diagnosis problem. Section 3 details the proposed methodology, including GraphSage and the attention-enhanced GraphSage framework for aircraft sensor fault diagnosis. Section 4 presents experimental results and analysis and Section 5 summarizes the main conclusions of this work.

## 2. Problem Definition

We start with air data evolution equations in defining the aircraft sensor fault detection and classification problem [34,35]:

(1) $$\left\{\begin{array}{c} \begin{array}{cc} \dot{V}= & \left(G_{x}-gS_{\theta }\right)C_{\alpha }C_{\beta }+(G_{y}+gS_{\phi }C_{\theta )}S_{\beta }\\ & +\left(G_{z}+gC_{\phi }C_{\theta }\right)S_{\alpha }C_{\beta }\\ \dot{\alpha }= & \left(-G_{x}S_{\alpha }+G_{z}C_{\alpha }+gC_{\phi }C_{\theta }C_{\alpha }+gS_{\theta }S_{\alpha }\right)/VC_{\beta }\\ & +w_{y}-\left(w_{x}C_{\alpha }+w_{z}S_{\alpha }\right)S_{\beta }/C_{\beta }\\ \dot{\beta }= & [-\left(G_{x}-gS_{\theta }\right)C_{\alpha }S_{\beta }+\left(G_{y}+gS_{\phi }C_{\theta }\right)C_{\beta }\\ & -\left(G_{z}+gC_{\phi }C_{\theta }\right)S_{\alpha }S_{\beta }]/V+w_{x}S_{\alpha }-w_{z}C_{\alpha } \end{array} \end{array}\right.$$

where the trigonometric functions *sin* and *cos* are abbreviated as $S_{*}$ and $C_{*}$; the variables *V*, α, and β represent the velocity, angle of attack, and sideslip angle, respectively; *g* denotes the gravitational acceleration; $\left\{w_{x},w_{y},w_{z}\right\}$, {ψ,θ,ϕ}, and $\left\{G_{x},G_{y},G_{z}\right\}$ represent the body-axis angular velocity components, aircraft Euler angles, and body-axis load factor components, respectively. The body-axis load factor components $\left\{G_{x},G_{y},G_{z}\right\}$ represent the total acceleration (including both gravitational and inertial effects) acting on the aircraft along its body-fixed coordinate axes, normalized by the gravitational acceleration.

In Equation (1), angular velocities and Euler angles of the aircraft are coupled as follows:

(2) $$\left\{\begin{array}{c} \begin{array}{cc} \dot{\psi } & =w_{y}S_{\phi }/C_{\theta }+w_{z}C_{\phi }/C_{\theta }\\ \dot{\theta } & =w_{y}C_{\phi }-w_{z}S_{\phi }\\ \dot{\phi } & =w_{x}+w_{y}S_{\phi }S_{\theta }/C_{\theta }+w_{z}C_{\phi }S_{\theta }/C_{\theta } \end{array} \end{array}\right.$$

And aircraft motion equations are written as follows:

(3) $$\left\{\begin{array}{c} \begin{array}{cc} \dot{x} & =uC_{\theta }C_{\psi }+v\left(S_{\theta }S_{\phi }C_{\psi }-C_{\phi }S_{\psi }\right)\\ & +w(S_{\theta }C_{\phi }C_{\psi }+S_{\phi }S_{\psi })\\ \dot{y} & =uC_{\theta }S_{\psi }+v\left(S_{\theta }S_{\phi }S_{\psi }+C_{\phi }C_{\psi }\right)\\ & +w(S_{\theta }C_{\phi }S_{\psi }-S_{\phi }C_{\psi })\\ \dot{z} & =-uS_{\theta }+vS_{\phi }C_{\theta }+wC_{\phi }C_{\theta } \end{array} \end{array}\right.$$

wherein the velocity component {u,v,w} expressed in the body axes are as follows:

(4) $$\left\{\begin{array}{c} \begin{array}{cc} u & =VC_{\alpha }C_{\beta }\\ v & =VS_{\beta }\\ w & =VS_{\alpha }C_{\beta } \end{array} \end{array}\right.$$

The flight state monitoring system relies on multiple onboard sensors. The primary sensor systems include air data sensors (ADSs) for measuring flight parameters V,α,β, and inertial measurement unit (IMU) for obtaining motion parameters $w_{x},w_{y},w_{z}$, ψ,θ,ϕ, and $G_{x},G_{y},G_{z}$. The aircraft’s position x,y,z is tracked through GPS signals. This research specifically addresses the fault detection and classification challenges in ADS and IMU systems.

While other studies typically employ model-based approaches to analyze the dynamics and kinematics represented in Equations (1)∼(4), our approach involves modeling the fault detection and classification problem as a mapping process. We utilize a GNN-based learning method to capture and explore the interrelationships within the sensor measurement data, facilitating the detection and classification of potential sensor faults.

## 3. Method Details

### 3.1. GraphSage

The graph neural network (GNN) is a deep learning algorithm specifically designed for analyzing graph-structured data [36]. In graph-structured data [37], information is represented as a network graph G = (V, E, A), where V represents the nodes, E represents the edges connecting them, and A denotes the weighted adjacency matrix. Unlike traditional data structures that treat samples independently, graph-structured data explicitly model the dependencies and connections between entities, enabling effective analysis of complex, interconnected systems.

As one of the most prominent variants of GNN, graph convolutional networks (GCNs) extend the success of convolutional neural networks (CNNs) to graph-structured data by redefining the convolution operation in the graph domain. Similar to how CNNs process local information in grid-like data, GCNs operate on each node by aggregating and transforming features from its local neighborhood, effectively capturing both node attributes and graph topology. This localized feature learning mechanism enables GCNs to learn hierarchical representations of nodes while preserving the structural information encoded in the graph, making it particularly effective for tasks such as node classification and graph representation learning.

**Figure 1:**
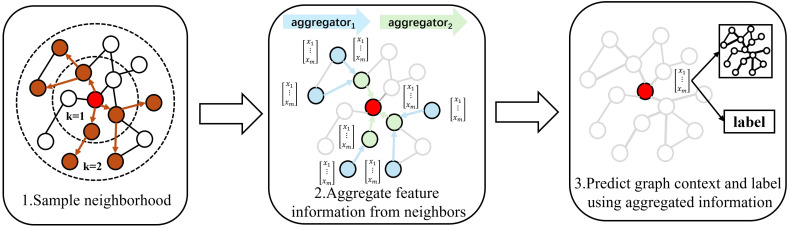
The algorithm flow of GraphSage (colored arrows represent the relationships between nodes).

However, GCNs have problems of poor scalability and flawed training methods. In order to further improve the training efficiency of the model, GraphSage has been proposed, which is an iterative graph neural network algorithm that aggregates neighboring nodes. GraphSage aggregates the neighborhood information of neighboring nodes by sampling them, reducing the computational consumption of graph neural networks during training and testing, which makes GraphSage highly adaptable to the distributed training of large-scale graph data. GraphSage aggregates the information of nodes and their neighbors in each layer of aggregation functions to obtain the feature vectors of the next layer. The algorithm flow is shown in Figure 1.

The GraphSage algorithm mainly includes two processes: graph sampling and graph aggregation.

(1) Graph Sampling: GraphSage uses the relationship between nodes to connect information, and adopts neighborhood sampling to control the growth rate of nodes when subgraphs diverge, keeping the size of subgraph nodes below the factorial level and freeing up space for model construction.

(2) Graph Aggregation: The information obtained after graph sampling is passed through multiple layers of aggregation functions, where the information of adjacent nodes is continuously fused. The fused features are used to represent the predicted node labels.

The sampling and aggregation process of GraphSage can be represented by the following formula:

(5) $$\begin{array}{c} h_{v}^{\left(l+1\right)}=\sigma (W\cdot \mathrm{MEAN}\left(\left\{h_{v}^{l}\right\}\cup \left\{h_{u}^{l},\forall u\in N_{\left(l+1\right)}\left(v\right)\right\}\right) \end{array}$$

(6) $$\begin{array}{c} h_{v}^{\left(l+1\right)}=\sigma \left(W\cdot [h_{v}^{l}|h_{N(v)}^{\left(l+1\right)}]\right) \end{array}$$

(7) $$\begin{array}{c} h_{v}^{{\left(l+1\right)}^{nor}}=\frac{h_{v}^{\left(l+1\right)}}{{∥h_{v}^{\left(l+1\right)}∥}_{2}} \end{array}$$

where *l* is the number of layers in the aggregation function; N(v) represents the result of uniformly sampling the first-order connected nodes of node *v*; *W* is the weight matrix; [·∣·] means the concatenation of two variables; σ is the activation function. Equation (7) is the normalization process for the data.

### 3.2. The Proposed Model

**Figure 2:**
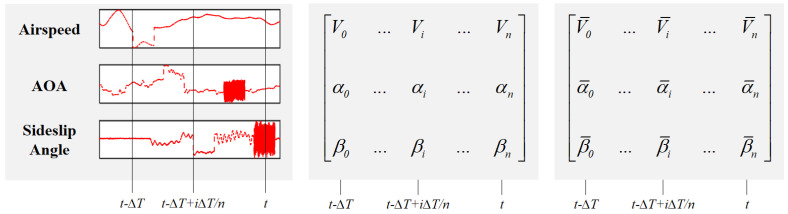
Illustrative depictions of the SDI are presented. On the (**left**), flight records (*V*, α, β) are collected from real or simulated flights. Faults are intentionally introduced into the data, and a time window (ΔT) is applied to segment the data, which is subsequently down-sampled to 1 Hz. In the (**middle**), the segmented and down-sampled data are concatenated into a matrix. On the (**right**), this matrix undergoes linear normalization, ensuring values fall within the 0∼1 range along each row. The SDI stacking procedure encompasses all 12 flight states and 3 load factors, resulting in the creation of a 15×N SDI grayscale image, where *N* represents the number of samples determined by ΔT.

**Figure 3:**
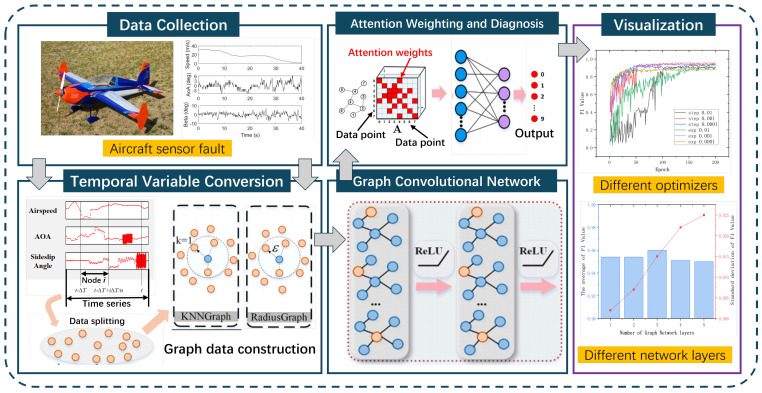
The proposed GraphSage-Attention model.

This section presents our proposed model for aircraft sensor fault diagnosis. We first transform the raw sensor measurements into SDIs (Sensor Data Images), which effectively capture the temporal and cross-sensor relationships in the data (Figure 2). Then, we construct graph structures from these SDIs using two complementary approaches: KNN and Radius graphs. Finally, we present an attention-enhanced GraphSage framework that processes these graph structures for fault detection and classification. The overall architecture of our proposed model is shown in Figure 3, which consists of two key components: (1) graph construction from SDIs through KNN and Radius approaches, and (2) an attention mechanism integrated into the GraphSage framework to enable adaptive feature learning. These components work collaboratively to form a comprehensive feature learning architecture, which will be detailed in the following.

**Figure 4:**
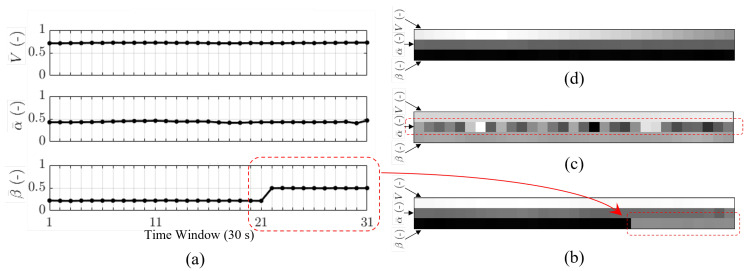
(**a**) The sideslip angle ($\bar{\beta }$) drift fault emerges during the time interval from 21 s to 31 s; (**b**) the fault is visually represented as a protruding strip within the SDI; (**c**) an illustration of extra noise in the AOA sensor ($\bar{\alpha }$) on the SDI, with the middle row exhibiting the presence of noise; (**d**) for the no-fault case, the SDI, where each row and column evolves in accordance with Equations (1)∼(3), does not exhibit any abnormal regions in the image.

The Imagefication-based fault detection and classification approach, as elucidated in [38], is contingent upon the consolidation of sensor data images (SDIs), as illustrated in Figure 2. This process involves the accumulation of measurement data pertaining to all flight states and load factors through simulated or real flight operations. Subsequently, faults are introduced into the measurement data from the ADS and IMU. These data are then subjected to normalization and arranged into a 2D matrix. In this matrix, each row represents the data sequence corresponding to a specific sensor (totaling 15 sensors for 12 flight states and 3 load factors), and each column corresponds to the measurement data of each sensor at a specific point in time (with the number of data points determined by the sampling frequency of 1 Hz and the selected time window ΔT). The dimensions of the SDI are established as 15×N, with values within each index subjected to normalization. Consequently, the SDI transforms the data into the format of a grayscale image sized 15×N, as depicted in Figure 4. In this representation, sensor faults manifest as abnormal regions within the grayscale image, similar to protruding stripes or oscillating noise patterns. This process effectively converts the original sensor fault detection and classification problem into an abnormal object detection and classification task performed on the grayscale SDI. In this paper, the input is an SDI, and the output comprises the fault labels ).

**Figure 5:**
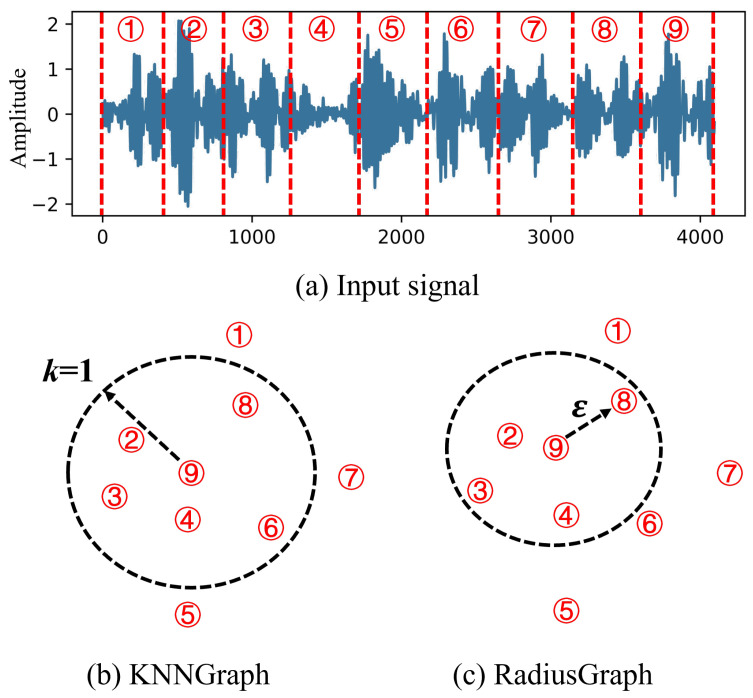
The calculation processes of the KNNGraph and RadiusGraph (numbers 1–9 represent different data segments on the time series).

In KNNGraph, for each node, the algorithm identifies the *k* closest neighbors, which can be represented as the neighbors of node $x_{i}$ (Figure 5).

(8) $$\begin{array}{c} \mathrm{Ne}\left(x_{i}\right)=\mathrm{KNN}\left(k^{\prime },x_{i},\Psi \right)\; \end{array}$$

where KNN(·) identifies the top-*k* closest neighbors of node $x_{i}$ in the set Ψ, and $k^{\prime }$ is set to 5 in this paper. Here, $\Psi =\left[x_{i+1},x_{i+2},\ldots ,x_{i+m}\right]$ refers to a subset containing *m* samples, and Ne denotes the neighbors associated with node $x_{i}$.

The edge weight between every node in the KNNGraph can be calculated using a Gaussian kernel weight function, defined as follows:

(9) $$\begin{array}{c} e_{ij}=\exp\left(-\frac{{∥\left(x_{i},x_{j}\right)∥}^{2}}{2\zeta^{2}}\right),\;x_{j}\in \mathrm{Ne}\left(x_{i}\right)\; \end{array}$$

where $e_{ij}$ represents the edge weight connecting node $x_{i}$ and node $x_{j}$, and ζ indicates the bandwidth of the Gaussian kernel.

In RadiusGraph, cosine similarity serves to evaluate the distance between samples, with a defined threshold ϵ. An edge is created between two nodes if their cosine similarity exceeds this threshold, allowing for the identification of neighbors for node $x_{i}$.

(10) $$\begin{array}{c} \mathrm{Ne}\left(x_{i}\right)=\epsilon ⊖-\mathrm{radius}\left(x_{i},\Psi \right),\;\;\mathrm{if}\;\;\epsilon ⊖-\mathrm{radius}\left(x_{i},\Psi \right)>\epsilon \end{array}$$

where ϵ⊖−radius(·) computes the cosine similarity between node $x_{i}$ and the node in set Ψ, identifying the neighbors of $x_{i}$. In this paper, the threshold ϵ is set to 0.

On the other hand, a specialized attention mechanism is introduced into the original GraphSage model to adaptively learn the attention weights between node features, in order to better capture important features and achieve fault diagnosis. Given the node feature x and edge index V, the calculation steps of the attention mechanism are as follows:

(11) $$\begin{array}{c} \alpha_{ij}=\mathrm{Attention}\left(x_{edge_{ij}}\cdot a_{w}\right) \end{array}$$

where $a_{w}$ is the learned attention weight, and $x_{edge_{ij}}$ is the feature extracted from the node pairs, representing the connections between node *i* and its neighbor *j*.

In order to make the contributions of each node comparable to each other, the obtained attention weights are normalized:

(12) $$\begin{array}{c} \alpha_{i}=\frac{\alpha_{ij}}{\sum_{j\in N(i)}\alpha_{j}} \end{array}$$

where N(i) represents the set of neighbors of node *i*, with the value of $\sum_{j\in N(i)}\alpha_{j}$ being 1.

Finally, the fused node features are aggregated and weighted by the following:

(13) $$\begin{array}{c} h_{i}^{\mathrm{out}\;}=\sum_{j\in N(i)}\alpha_{ij}\cdot e_{ij} \end{array}$$

where $h_{i}^{\mathrm{out}\;}$ is the updated node feature representation, which is weighted and summed by multiplying the edge of each neighbor $e_{ij}$ by the corresponding attention score αij.

By introducing the attention mechanism, the model implements an adaptive neighbor selection strategy through learnable attention weights. This mechanism enables the model to assign different importance scores to neighboring nodes during the information aggregation process, allowing selective emphasis on node features based on their relative importance in the local structure.

## 4. Experimental Details

### 4.1. Experimental Environment

The experimental platform operates on Microsoft Windows 10 with an eight-core processor (16 threads) and GTX 3090 graphics card, as detailed in Table 1. The implementation uses Python 3.8 and PyTorch 1.9.0 for constructing and training the proposed model.

### 4.2. Dataset

Aircraft sensors are responsible for monitoring vital aircraft parameters such as airspeed, attitude angles, and load factors, etc. Extensive literature highlights numerous catastrophic aircraft accidents attributed to sensor faults [2,39]. Nevertheless, the acquisition of aviation sensor data remains both cost-prohibitive and challenging. Consequently, the development of fault detection and classification technology (FDC) for diverse types of sensors between different aircraft is of paramount importance.

The dedicated database, as detailed in Table 2, has been meticulously curated. This database comprises a comprehensive collection of both simulated and real-flight data sourced from four distinct aircraft platforms, namely, a large cargo airplane designated as Y [40], a passenger aircraft designated as B_1_ [41], a general aviation aircraft designated as D [42,43], and a unmanned simulated aircraft designated as F [44]. These datasets encompass three diverse flight conditions, encompassing high and low altitudes for cruise, as well as low-altitude flight under manual control. Additionally, the database incorporates various control modes, spanning human pilot (manual) control and automated control laws (auto-pilot, AP).

For simulation data, dryden atmospheric disturbances [45] were intentionally introduced to perturb the flight states, followed by the addition of measurement noise to generate the noise-corrupted data. The measurement noise is modeled with an assumption of Gaussian distribution. The standard deviations for the noise associated with each sensor are detailed in Table 3 [35].

Previous research has extensively examined a range of sensor faults, including but not limited to ramp bias, oscillations, and drift. Notably, many aviation accidents have been attributed to the Pitot tube becoming obstructed, leading to airspeed-related issues. Consequently, drift faults (manifesting as measurement loss) are taken into consideration for airspeed sensors. As for angle of attack (AOA) and sideslip angle sensors, potential issues may arise from deflection vanes getting stuck or perturbed by external atmospheric conditions, giving rise to drift (constant bias) and additional noise faults. In the context of IMU sensors, the fault models adhere to the approaches outlined in [8,35,46]. As detailed in Table 4, a total of nine distinct fault cases are explored, each case’s magnitude being specified in accordance with [43]. By combining the normal data, we have established an aircraft sensor fault detection database that encompasses 10 categories.

The implementation of aircraft sensor faults follows an additive approach, where the “clean” data (Case 0 in Table 4) are obtained from real flight or simulations. Subsequently, sensor faults are introduced into the measurement data. Following the methodology outlined in [43], this injection process is executed in a randomized manner. Specifically, for every 60 s interval within the data, fault cases occur randomly at unpredictable moments, with their duration (also randomized) not exceeding 60 s.

### 4.3. Evaluation Metrics

For the two evaluation metrics (accuracy and F1), Table 5 depicts the correspondence between the predictions of the proposed model and the true labels.

Expanding to the multi-classification tasks (taking *n* classes as an example), the values of $\vec{TP}$ (True Positive), $\vec{FP}$ (False Positive), $\vec{FN}$ (False Negative), and $\vec{TN}$ (True Negative) are *n*-dimensional vectors, where *n* represents the number of classes in the dataset (in this study, *n* = 10).

(14) $$\vec{TP}=\left|\begin{array}{c} TP_{0}\\ TP_{1}\\ \cdots \\ TP_{n-1} \end{array}\right|$$

In Equation (14), each dimension of the vector represents a specific value for a particular class. Assuming there are *M* samples, for a specific sample *S*, the true label for the $k^{th}$ ($k\in \left[0,n-1\right]$) specific class is denoted as $L_{k}$, and the predicted class is denoted as $P_{k}$.

(15) $$\begin{array}{c} L_{k}=1,P_{k}=1,\;\mathrm{then}\;{TP}_{k}=1\\ L_{k}=0,P_{k}=1,\;\mathrm{then}\;{FP}_{k}=1\\ L_{k}=0,P_{k}=0,\;\mathrm{then}\;{TN}_{k}=1\\ L_{k}=1,P_{k}=0,\;\mathrm{then}\;{FN}_{k}=1\; \end{array}$$

The ${\vec{TP}}_{S}$ for sample *S* is formed by combining the aforementioned results into a vector.

(16) $${\vec{TP}}_{S}=\left|\begin{array}{c} TP_{0}\\ TP_{1}\\ \cdots \\ TP_{n-1}\; \end{array}\right|$$

The final result of $\vec{TP}$ is a vector obtained by summing the results for *M* samples.

(17) $$\vec{TP}={\vec{TP}}_{0}+{\vec{TP}}_{1}+\cdots +{\vec{TP}}_{M-1}$$

Using the same calculation steps and methods as the above, $\vec{FP}$, $\vec{FN}$, and $\vec{TN}$ can be obtained. The calculation formulas for evaluation metrics (acc, prec, rec, and f1) are as follows:

(18) $$accuracy=\left|\frac{\vec{TP}+\vec{TN}}{\vec{TP}+\vec{TN}+\vec{FN}+\vec{FP}}\right|$$

(19) $$F1=\left(2\times \frac{prec\times rec}{prec+rec}\right)$$

### 4.4. Superparameter Comparison Experiment

The 294,144 sample data were collected in the experiment, and will be segmented according to different segmentation lengths in the future. Divide the data into training data and testing data in a 9:1 ratio. The default initialization parameters for model training were as follows: the optimizer was set to SGD with a learning rate of step 0.01, the number of layers in the graph network was set to 3, the batch size was set to 6, and the length of the segmented samples was set to 128. In order to obtain optimized hyperparameter combinations, this paper conducted comparative experiments on the optimizer, graph network layers, and batch size.

**Figure 6:**
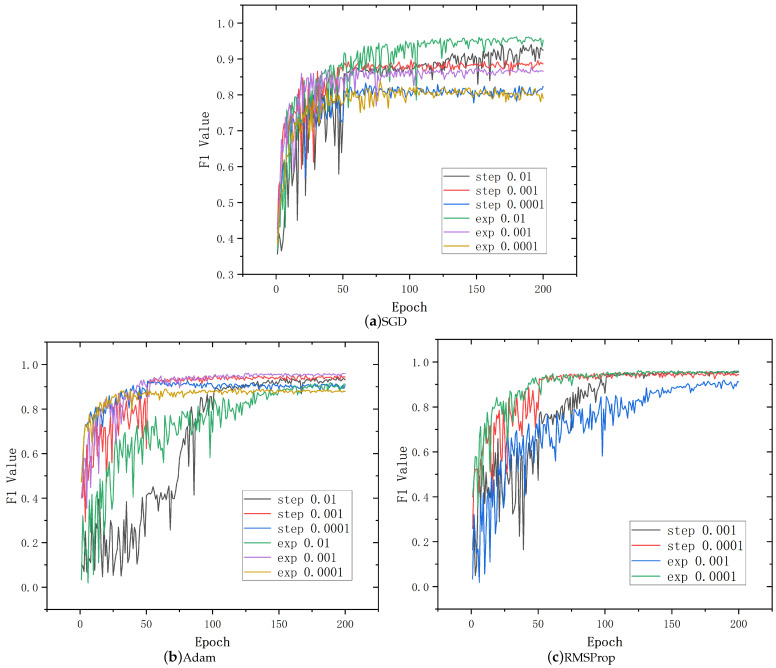
Comparative experimental results of optimizer and learning parameters.

Firstly, in order to achieve higher fault diagnosis rates and ensure smoother network training, this paper conducted comparative experiments on three sets of optimizers under different learning rates and strategies. The experimental results are shown in Table 6 and Figure 6. The F1 value was used to evaluate the results of different optimizers, which provided a more comprehensive assessment of model performance. The experimental results indicated that Adam and RMSProp optimizers had higher accuracy in fault diagnosis. However, it is worth noting that RMSProp had a faster convergence speed compared to Adam. Therefore, in the experiment of this paper, the optimizer with RMSProp with exp 0.0001 was chosen as the optimal choice.

**Figure 7:**
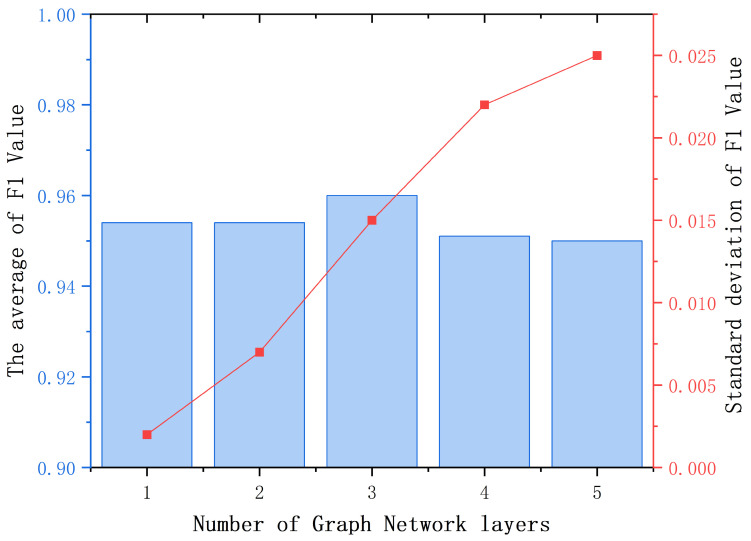
Comparative experiment results of graph network layers.

**Figure 8:**
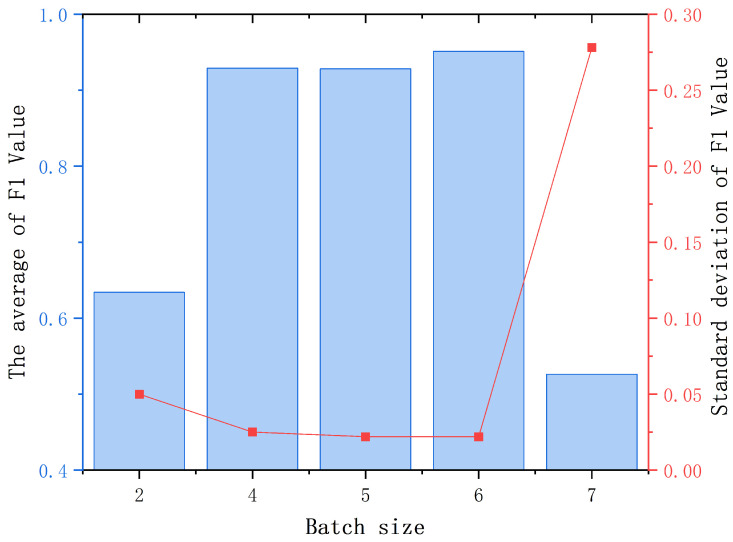
Comparative experiment results of batch size.

In addition, comparative experiments were conducted on the number of layers and batch size of graph networks under different hyperparameter values, and the results are shown in Table 7 and Table 8, respectively. In both hyperparameter experiments, this study conducted five repeated experiments to ensure the reliability of the results. This article visualizes the mean and variance in the statistical results, as shown in Figure 7 and Figure 8, respectively. The experimental results indicate that setting the number of layers in the graph network to 3 and the batch size to 6 will result in more optimized fault diagnosis results.

### 4.5. Ablation Experiment

In order to verify that the attention weighting proposed in this paper is beneficial for the network to perform fault diagnosis tasks, ablation experiments were conducted in this paper. The results are shown in Table 9. Perform fault diagnosis tasks using models that eliminate attention and models with attention, respectively, under different sample lengths and composition methods. When the sample length was set to 64, 128, and 512, adding attention effectively improved the diagnostic accuracy of the model, reaching up to 7.4%. However, when the sample length was set to 1024, the diagnostic accuracy of the model decreased after adding attention. There are two main reasons. On the one hand, increasing the sample length reduces the number of samples, and the addition of attention makes the model more complex, which can easily lead to overfitting problems. On the other hand, an increase in sample length makes the model more susceptible to the influence of features other than important information, and the addition of attention strengthens this trend.

### 4.6. Comparison with Other Advanced Experiments

To verify the effectiveness of the proposed model, this paper compared the diagnostic results with other advanced graph network models, including Multi-Layer Perceptron (MLP), Graph Convolutional Network (GCN) [47], Higher-Order Graph Convolutional Network (HoGCN) [48], ChebyNet [49], Simplifying Graph Convolutional Network (SGCN) [50], Graph Attention Network (GAT) [51], and Graph Isomorphism Network (GIN) [52]. The experiment for each model was repeated four times to ensure the reliability of the results. The experimental results (Table 10) indicate that the composition of Radius will be more conducive to improving the diagnostic accuracy of most models. In addition, in sharp contrast to other models, the model proposed in this paper has the highest fault diagnosis performance, which verifies the effectiveness of the proposed model.

### 4.7. Actual Flight Data Testing

**Figure 9:**
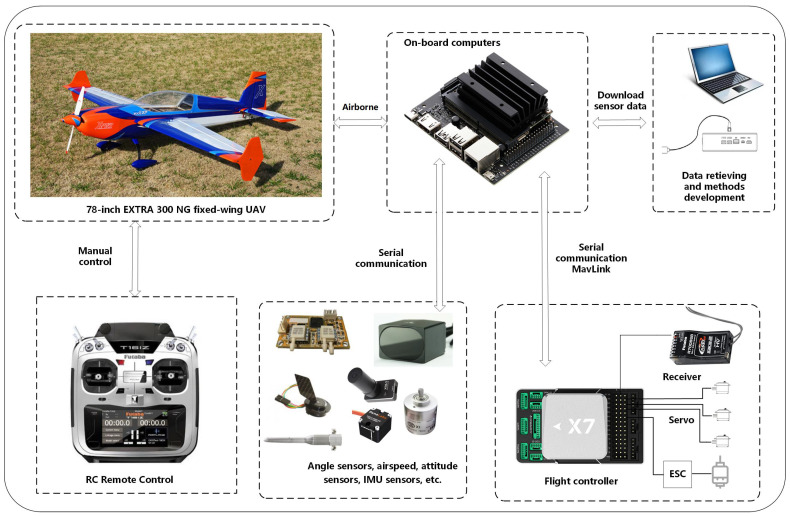
Configurations of the UAV adopted in this paper.

**Figure 10:**
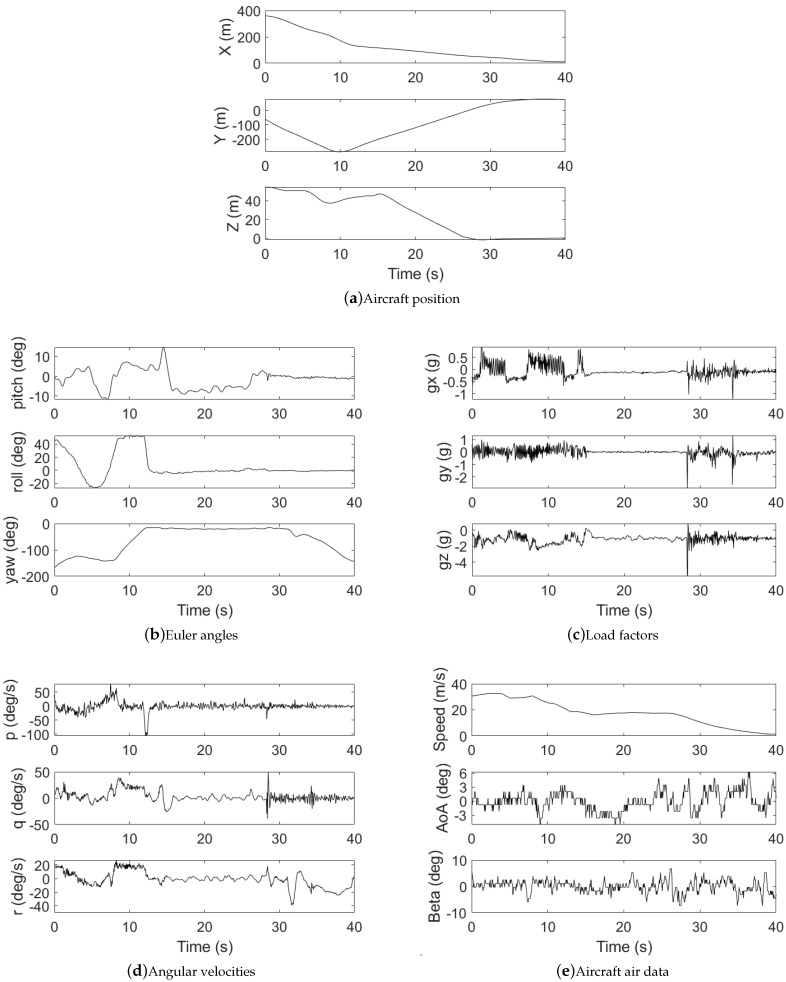
An illustrative plot of the real-flight data.

A 78-inch EXTRA 300 NG fixed-wing UAV equipped with CUAV X7+ PRO flight control (CUAV, Guangzhou, China) was used for data collection, as shown in Figure 9. The UAV integrates CUAV C-RTK 9P GPS, SMV-1 Hall principle angle measuring vane, and ADM800 altitude/airspeed meter. Manual flight tests were conducted at low altitude (below 100 m), collecting all 15 SDI variables (3 position coordinates, 3 Euler angles, 3 load factors, 3 angular velocities, and 3 air data states) through onboard sensors. An illustrative plot of the flight data is shown in Figure 10.

A total of 285,572 real-flight data points were obtained through fault injection and data augmentation (linear interpolation was used to align the measurement data of different sensors to ensure the integrity of stacked images). To assess the model’s performance comprehensively, accuracy and F1 value were selected as the evaluation metrics. F1 value, which considers the trade-off between precision and recall, provides a balanced evaluation.

Ultimately, considering detection accuracy, F1 value, model testing time, and comprehensive evaluation indicators, the experimental results are shown in Table 11. The calculation formula for comprehensive evaluation indicators (CEIs) is as follows:

(20) $$CEI=N\left(\vec{A}\right)/N\left(\vec{T}\right)$$

where $\vec{A}$ represents the vector composed of F1 Value, $\vec{T}$ represents the vector composed of Time, and *N* represents the maximum and minimum normalization operation. If the value in the $\vec{T}$ is 0, then the value is equal to 0.001.

The results on actual flight data are shown in Table 11. The ArcNet and MSAE algorithms lack sufficient extraction of coupling information between different signal channels, resulting in a noticeable decrease in accuracy compared to other algorithms. The table shows that CNN+XGBoost and CNN+SVM, although using XGBoost and SVM as classifiers, are sensitive to noise and outliers, leading to reduced accuracy and increased testing time. While the Modified CNN reduces testing time, it comes with the risk of sacrificing accuracy. The CNN+LSTM model, which considers both local and global features of the signals, achieved improved accuracy compared to the original convolutional neural network while further reducing testing time. The deep neural network models demonstrated further improvements in testing accuracy, but their large parameter sizes (all exceeding $10^{7}$) and high computational complexity resulted in testing times that exceeded expectations, limiting their applicability in certain scenarios such as edge computing. The proposed model presented in this paper attained superior performance, achieving the highest accuracy while also considering the algorithm’s runtime. This consideration enhances the model’s application performance, making it more suitable for practical implementation.

## 5. Conclusions

This work proposes an improved GraphSage fault diagnosis method that integrates an attention mechanism to solve the problems of insufficient data representation and insufficient information feature mining in traditional deep learning methods for aircraft sensor fault diagnosis. The method proposed in this paper can utilize the potential information between data and improve the model’s fault identification through the attention mechanism. This work conducted a large number of experiments, first discussing the influence of optimizers, graph network layers, and batch size parameters on the diagnostic results of the model by controlling variables. Secondly, the impact of attention on improving model performance was verified through ablation experiments. Finally, through comparative experiments with other advanced methods, it was verified that the method proposed in this paper can achieve the highest diagnostic accuracy. In addition, the fault diagnosis performance of the model has been validated on real-flight data, and it has a fast calculation speed. In future work, we will further explore the application of graph network technology in complex data representation, especially in aircraft fault data.

## Figures and Tables

**Table 1:** Hardware configurations in the experiments.

Component	Model
CPU processor	11th Gen Intel (R) Core (TM) i9-11900K
Memory	32 GB
Graphics card model	NVIDIA GeForce RTX 3090
Graphics card memory	40 GB

**Table 2:** Overview of aircraft sensor fault database used in this paper.

Aircraft	Configuration Overview	Quality	Span	Data Sources	Flight Condition	Data Duration
Y	Military transport aircraft	41 t	38 m	Flight simulation	Low altitude, cruise, AP	295 min
B1	Civil aviation airliner	174 t	59.6 m	Flight simulation	Low altitude, free flight, manual	151 min
D	General aviation aircraft	3.1 t	19.8 m	Flight simulation	High altitude, cruise, AP	162 min
F	Unmanned simulated aircraft	-	-	Real flight	-	-

**Table 3:** Sensor noises used in the database.

Sensor	Standard Deviation	Unit
$V_{m}$	0.1	[m/s]
${\left\{\alpha ,\beta \right\}}_{m}$	0.1	[deg]
${\left\{G_{x},G_{y},G_{z}\right\}}_{m}$	0.01	[g]
${\left\{p,q,r\right\}}_{m}$	0.01	[deg/s]
${\left\{\psi ,\theta ,\phi \right\}}_{m}$	0.01	[deg]
${\left\{x,y,z\right\}}_{m}$	1	[m]

**Table 4:** Aircraft sensor fault cases adopted in this paper.

Case	Sensor	Fault Type	Magnitude *
9	${\left\{G_{x},G_{y},G_{z}\right\}}_{m}$	extra noise	0.1∼0.3 g
8	${\left\{w_{x},w_{y},w_{z}\right\}}_{m}$	extra noise	5∼10°/s
7	${\left\{G_{x},G_{y},G_{z}\right\}}_{m}$	drift	±(0.1∼0.3 g)
6	${\left\{w_{x},w_{y},w_{z}\right\}}_{m}$	drift	±(5∼10°/s)
5	$\beta_{m}$	extra noise	5∼10°
4	$\beta_{m}$	drift	±(5∼10°)
3	$\alpha_{m}$	extra noise	5∼10°
2	$\alpha_{m}$	drift	±(5∼10°)
1	$V_{m}$	drift	−(50∼100%)
0	clean measurement with noises and disturbances, no fault		

* Noise standard deviation and drift values defined in this column.

**Table 5:** Confusion matrix of predictions and labels.

	Predictions (Positive)	Predictions (Negative)
Labels (Positive)	TP	FN
Labels (Negative)	FP	TN

**Table 6:** Comparative experiment of optimizer and learning parameters.

	Parameter	Step 0.01	Step 0.001	Step 0.0001	Exp 0.01	Exp 0.001	Exp 0.0001
Optimizer							
SGD		0.924	0.887	0.824	0.953	0.865	0.79
Adam		0.93	0.943	0.907	0.917	0.936	0.878
RMSprop		-	0.955	0.944	-	0.912	0.959

**Table 7:** Comparative experiment results of graph network layers.

	Layers	1	2	3	4	5
Times						
1		0.954	0.94	0.975	0.952	0.96
2		0.952	0.955	0.955	0.966	0.972
3		0.958	0.957	0.968	0.912	0.918
4		0.952	0.956	0.934	0.952	0.922
5		0.956	0.962	0.97	0.975	0.977
Average of F1		0.954	0.954	0.96	0.951	0.95
Standard deviation of F1		0.002	0.007	0.014	0.022	0.025

**Table 8:** Comparative experiment results of batch size.

	Batch	2	4	5	6	7
Times						
1		0.67	0.947	0.965	0.967	0.158
2		0.69	0.905	0.911	0.969	0.663
3		0.663	0.899	0.937	0.937	0.523
4		0.571	0.928	0.9	0.914	0.964
5		0.575	0.967	0.928	0.966	0.324
Average of F1		0.634	0.929	0.928	0.951	0.526
Standard deviation of F1		0.0504	0.0255	0.0225	0.0218	0.278

**Table 9:** Ablation experimental results of attention.

	Sample Length	64	128	512	1024
Methods					
KNN	Without attention	0.892	0.848	0.941	0.943
	With attention	0.939	0.871	0.963	0.933
	Increase in F1	0.047	0.023	0.022	−0.010
Radius	Without attention	0.840	0.918	0.914	0.961
	With attention	0.914	0.974	0.958	0.951
	Increase in F1	0.074	0.056	0.044	−0.010

**Table 10:** Comparative experiments with other advanced models.

	MLP	GCN	HoGCN	ChebyNet	SGCN	GAT	GIN	GraphSage-Attention
KNN	0.864	0.503	0.907	0.807	0.493	0.899	0.886	0.928
	0.865	0.5	0.906	0.826	0.528	0.894	0.891	0.923
	0.865	0.515	0.906	0.815	0.494	0.898	0.887	0.925
	0.86	0.469	0.902	0.785	0.484	0.895	0.876	0.923
Average of F1	0.864	0.5	0.905	0.808	0.5	0.897	0.885	0.925
Radius	0.904	0.681	0.86	0.838	0.656	0.918	0.936	0.987
	0.905	0.714	0.862	0.845	0.69	0.922	0.937	0.988
	0.902	0.681	0.862	0.834	0.655	0.917	0.935	0.987
	0.902	0.688	0.86	0.833	0.661	0.918	0.935	0.987
Average of F1	0.903	0.691	0.861	0.838	0.666	0.919	0.936	0.987

**Table 11:** Experimental results of comparative methods.

	Methods	Accuracy	F1 Value	Time (s)	CEI
1	ArcNet [53]	60.94	56.22	0.2	0
2	MSAE [54]	67.19	64.04	0.64	2.25
3	CNN+XGBoost [55]	78.13	78.83	3.51	1.08
4	Modified CNN [56]	84.38	84.31	0.86	5.83
5	CNN+SVM [57]	85.94	86.05	6.79	0.73
6	CNN [58]	89.07	89.27	1.11	5.21
7	CNN+LSTM [59]	90.63	90.32	1.01	5.95
8	Transformer [60]	93.75	93.66	5.25	1.19
9	ADG-dCNN [61]	95.31	95.24	1.84	3.62
10	GraphSage-Attention	97.80	97.20	0.07	1000

## Data Availability

Data are available on request from the authors.

## Abbreviations

Terms	Full Name and Explanation
AOA	angle of attack
DNNs	deep neural networks
CNNs	convolutional neural networks
AEs	autoencoders
RNNs	recurrent neural networks
GNNs	graph neural networks
GCNs	graph convolutional networks
*V*	velocity
α	angle of attack
β	sideslip angle
*l*	the number of layers in the aggregation function
N(v)	the result of uniformly sampling the first-order connected nodes of node *v*
*W*	weight matrix
σ	activation function
KNN(·)	the top-*k* closest neighbors of node $x_{i}$ in the set Ψ
$e_{ij}$	the edge weight connecting node $x_{i}$ and node $x_{j}$
ζ	the bandwidth of the Gaussian kernel
$a_{w}$	the learned attention weight
$x_{edge_{ij}}$	the feature extracted from the node pairs
N(i)	the set of neighbors of node *i*
$h_{i}^{\mathrm{out}\;}$	the updated node feature representation
CEI	comprehensive evaluation indicators
